# Anti-breast cancer effects of dairy protein active peptides, dairy products, and dairy protein-based nanoparticles

**DOI:** 10.3389/fphar.2024.1486264

**Published:** 2024-11-13

**Authors:** Deju Zhang, Ying Yuan, Juan Xiong, Qingdong Zeng, Yiming Gan, Kai Jiang, Ni Xie

**Affiliations:** ^1^ Biobank, Shenzhen Second People’s Hospital, First Affiliated Hospital of Shenzhen University, Shenzhen, China; ^2^ Guangdong Key Laboratory for Biomedical Measurements and Ultrasound Imaging, National-Regional Key Technology Engineering Laboratory for Medical Ultrasound, School of Biomedical Engineering, Shenzhen University Medical School, Shenzhen, China; ^3^ Food and Nutritional Sciences, School of Biological Sciences, The University of Hong Kong, Pokfulam, Hong Kong SAR, China; ^4^ Hengyang Medical School, University of South China, Hengyang, Hunan, China; ^5^ Plant Science, School of Biological Sciences, The University of Hong Kong, Pokfulam, Hong Kong SAR, China; ^6^ Eastern Institute for Advanced Study, Eastern Institute of Technology, Ningbo, Zhejiang, China

**Keywords:** milk-derived peptides, breast cancer, nanoparticles, apoptosis, antibreast cancer

## Abstract

Breast cancer is the most frequently diagnosed and fatal cancer among women worldwide. Dairy protein-derived peptides and dairy products are important parts of the daily human diet and have shown promising activities in suppressing the proliferation, migration, and invasion of breast cancer cells, both *in vitro* and *in vivo*. Most of the review literature employs meta-analysis methods to explore the association between dairy intake and breast cancer risk. However, there is a lack of comprehensive summary regarding the anti-breast cancer properties of dairy protein-derived peptides, dairy products, and dairy protein-based nanoparticles as well as their underlying mechanisms of action. Therefore, the present study discussed the breast cancer inhibitory effects and mechanisms of active peptides derived from various dairy protein sources. Additionally, the characteristics, anti-breast cancer activities and active components of several types of dairy products, including fermented milk, yogurt and cheeses, were summarized. Furthermore, the preparation methods and therapeutic effects of various dairy protein-containing nanoparticle delivery systems for breast cancer therapy were briefly described. Lastly, this work also provided an overview of what is currently known about the anti-breast cancer effects of dairy products in clinical studies. Our review will be of interest to the development of natural anticancer drugs.

## 1 Introduction

Breast cancer represents a significant global health challenge, posing a major obstacle to the survival of patients and the augmentation of life expectancy worldwide (Chaudhari et al., 2024). In 2020, over 2.3 million women were diagnosed with breast cancer, resulting in 685,000 deaths (Arafat et al., 2023). Current treatments for breast cancer have considerable drawbacks. For instance, surgical methods may cause severe pain and compromise a woman’s physical integrity; radiation therapy may damage healthy cells and can thus lead to debilitation and shorter life expectancy; chemotherapy is not only cardiotoxic but also lead to drug resistance when administered for a long period, making breast cancer treatment particularly challenging (Anagnostopoulos and Myrgianni, 2009; Gärtner et al., 2009; Marquette and Nabell, 2012; Farhood et al., 2020; Wang and Tepper, 2021; Zhang T. Y. et al., 2023). Therefore, new approaches, especially those with minimal side effects, affordable costs, and high patient compliance, need to be developed urgently to cater to the expectations of patients undergoing breast cancer treatment and to reduce their economic and psychological burden.

The etiology of breast cancer is multifaceted, including a complex interplay of hereditary, dietary and environmental risk factors (Taleb et al., 2017). As suggested by several scholars, genetic determinants are responsible for 30%–40% of cancer cases, with the remaining 60%–70% ascribed to non-genetic environmental and lifestyle factors, including diet, exercise, obesity, geographic location, etc. (Keum and Giovannucci, 2019; Xiao et al., 2023). Among them, diet is one of the most modifiable aspects of lifestyle and is considered an effective strategy for breast cancer control (Arafat et al., 2023). There is substantial evidence suggesting that several food components, such as polysaccharides, milk proteins, polyphenols, and active peptides can markedly reduce the risk of breast cancer (Gu et al., 2020; Shin et al., 2020; Corso et al., 2021; Jabbari et al., 2022).

Dairy products are widely consumed across the globe and also an important part of dietary guidelines in various countries. The American Dietary Guidelines recommend that children and adolescents consume 2-3 cups of dairy products per day, the Chinese Dietary Guidelines suggest a milk intake of 300 g/day, and the French Dietary Guidelines recommend 2-3 servings of dairy products per day (Cámara et al., 2021). Furthermore, dairy products supply multiple nutrients for human metabolism, including macronutrients such as proteins and lipids, and micronutrients such as calcium, phosphorus, vitamin A, vitamin B, and riboflavin (Leischner et al., 2021). This comprehensive nutrient profile has attracted scholars to investigate the health benefits of dairy products.

**FIGURE 1:**
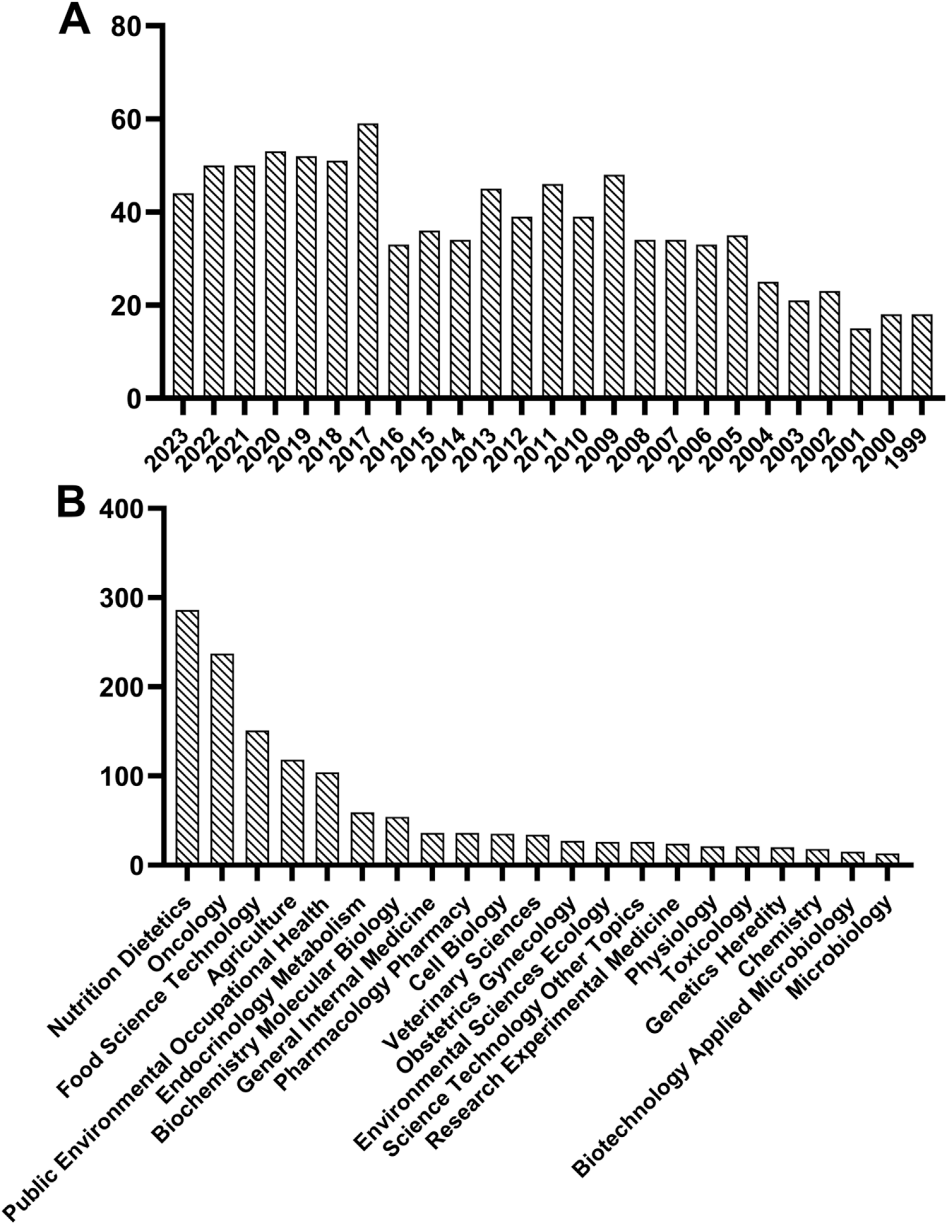
Statistics on dairy and breast cancer in web of science based on publication year **(A)** and research direction **(B)**.

Some studies have explored the association between dairy products and breast cancer risk. A case study involving 1,699 women showed that high consumption of dairy products among premenopausal women was linked to a reduced risk of breast cancer (Wajszczyk et al., 2021). Moreover, El-Din et al. reported that *L. kefiri* P-B1-fermented kefir significantly reduced tumor volume, enhanced breast cancer cell apoptosis and induced cell cycle arrest at G0/G1 phase in female Swiss albino mice injected with breast cancer cells (El-Din et al., 2020). These findings reveal a connection between dairy product intake and breast cancer progression. More than that, as shown in Figure 1A, research on breast cancer and dairy products has been on the rise over the past 25 years, according to the data retrieved from the Web of Science. In addition, the “Nutrition Dietetics,” “Oncology”, and “Food Science Technology” categories occupy a dominant role in the study of dairy products for breast cancer treatment (Figure 1B). The above evidence suggests that the importance of dairy products in the treatment of breast cancer is increasing, with numerous basic experimental and clinical studies demonstrating their positive effects. However, a review of recent progress in this field is still lacking.

**FIGURE 2:**
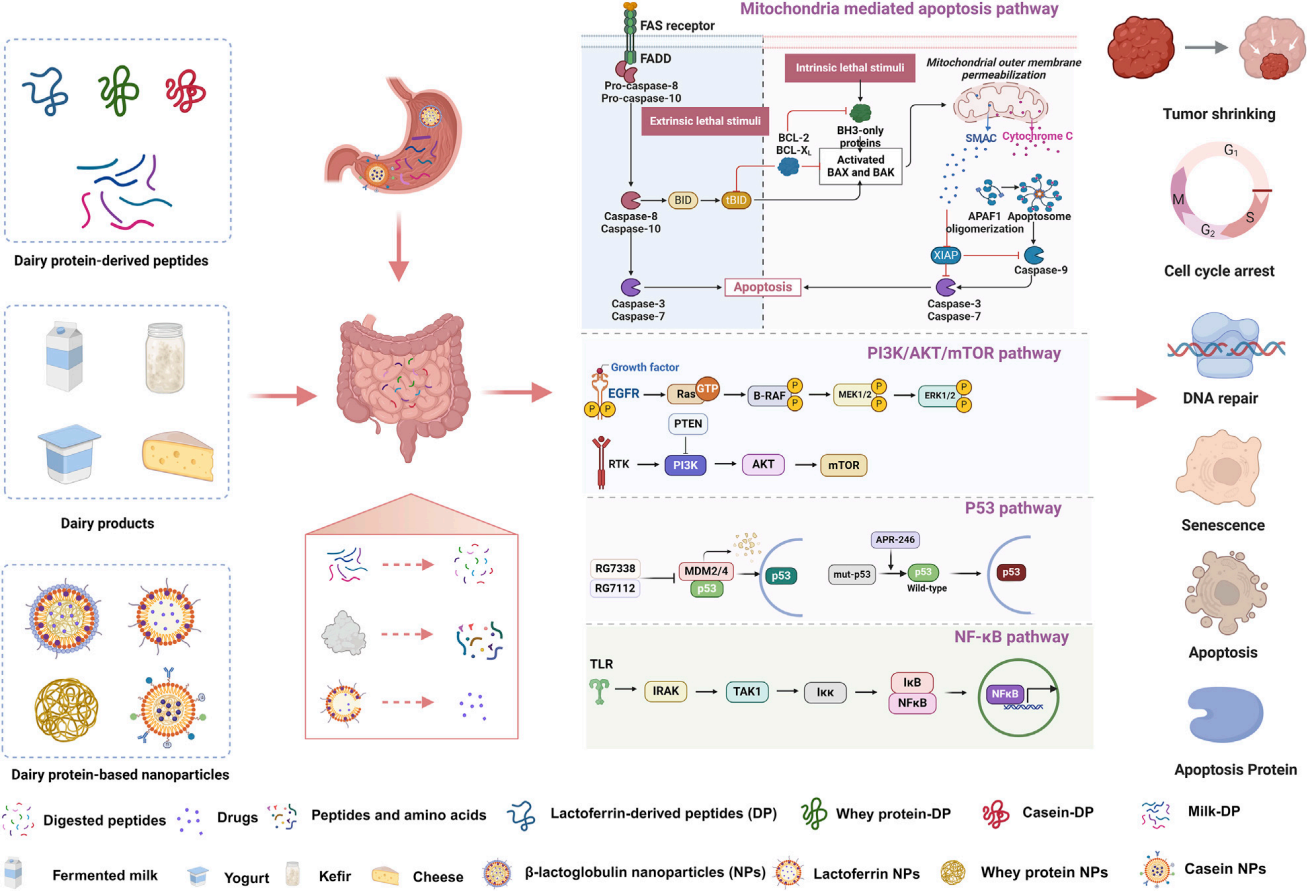
Anti-breast cancer effects and mechanisms of dairy peptides, dairy products and dairy protein-based nanoparticles.

To clarify the great promise of dairy protein-related products for breast cancer treatment, the following work was undertaken (Figure 2). First, we provided an overview of the sources, processing methods, peptide sequences and anti-breast cancer effects of different dairy protein-derived peptides and introduced the underlying mechanisms by which they exhibited anticancer effects. Second, we summarized the characteristics and anti-breast cancer effects of milk proteins, fermented milk, yogurt and cheese. Third, various nanoparticle delivery systems containing dairy proteins or dairy peptides, which were designed for breast cancer administration, were included in this study. Fourth, clinical effects of dairy peptides, dairy proteins and dairy products on breast cancer treatment were also discussed in detail. Our study will be of great interest to the deep processing and clinical application of dairy proteins and dairy products.

## 2 Anti-breast cancer peptides derived from dairy protein

Milk is naturally a whole food, composed of 87% water, 4% lipids and 9% water-soluble compounds (proteins, lactose, calcium, vitamin D, etc.) (Auestad and Layman, 2021). More importantly, milk is easily digestible and rich in nine essential amino acids, making it an important source of highly bioactive peptides. Consequently, the major protein constituents of milk, α-S1-casein, α-S2-casein, β-casein, κ-casein, the whey proteins α-lactalbumin, lactoferrin (LF), and β-lactoglobulin, have been widely used for the extraction of bioactive peptides.

### 2.1 Lactoferrin-derived anti-breast cancer peptides

LF, which contains approximately 690 amino acid residues and has a molecular weight of approximately 80 kDa, is a functional glycoprotein first isolated from cow’s milk by Sorensen in 1939 and first separated from human breast milk by Sorensen and Sorensen (1939), Johanson et al. (1960), Zhang Y. Q. et al. (2021). Typically, LF is odorless, readily soluble in water, and exhibits a certain level of structural stability. It can withstand temperatures of 56°C for several hours but is rapidly inactivated at temperatures above 80°C (Kowalczyk et al., 2022). LF consists of two homologous globular lobes, where a carboxyl (C) and an amino (N) end are linked by an α-helix. Each lobe contains two structural domains, designated as C1, C2, N1, and N2, and they form a β-sheet (Gruden and Ulrih, 2021). The structure of LF makes it highly flexible and can form different variants after transcription, translation, and modification (phosphorylation, acetylation, lipidation, ubiquitination, or glycosylation, etc.). This enables it not only to be serve as a polymeric material, but also to exhibit multiple biological activities *in vivo*, such as tumor growth inhibition, and antibacterial, free radical scavenging, antiviral, and anti-inflammatory properties (Gruden and Ulrih, 2021). In addition, LF has a high affinity for trivalent iron (K_D_ ∼ 10^–20^M), allowing it to regulate free iron in body fluids and perform multiple functions as an iron-binding glycoproteins (Zhang Y. Q. et al., 2021). LF with different iron saturation levels exhibits different biological functions. For instance, LF with higher iron content is thought to have antiviral capacity and bacteriostatic activity against both Gram-positive and Gram-negative bacteria, whereas LF with lower iron saturation could bind more iron, thereby reducing free radical damage caused by iron overload and exhibiting antioxidant capacity (Gruden and Ulrih, 2021; Zhang Y. Q. et al., 2021). Lactoferrin has a long history of dietary use and is classified as a Generally Recognized as Safe (GRAS) nutritional supplement by the U.S. Food and Drug Administration (Wakabayashi et al., 2006). Extensive studies in both animals and humans have shown that even in vulnerable populations with compromised immune systems or disease states, daily doses of lactoferrin ranging from 1.5 to 15 g for periods of 1 day–42 weeks have not demonstrated any significant toxicity-related outcomes in safety or tolerability assessments (Wakabayashi et al., 2006; Vishwanath-Deutsch et al., 2024). In recent years, as the relationship between dairy consumption and breast cancer risk reduction has become clearer, several scholars have also expressed interest in investigating the anti-breast cancer effects of LF-activated peptides.

Several possible anti-breast cancer peptides have been identified in LF such as FKCRRWQWRMKKLGAPSITCVRRAF, RRWQWRMKKLG, RRWQWR, RRWQWRMKKLG, FKARRWQWRMKKLGA, GEQELRKCNQWSGLSEGSVT, WSGLSEGSVTCSSASTTEDC, and RWQWRWQWR (Table 1). These LF-peptides inhibit the growth, adherence, and migration of breast cancer cells, promote cell death, induce cell cycle arrest, and lead to the overexpression of apoptosis-associated proteins, including caspase-3, -7, -8, and -9, thus hindering breast cancer progression. For instance, Fatemi et al. separated three peptides from human LF, namely GEQELRKCNQWSGLSEGSVT, WSGLSEGSVTCSSASTTEDC, and CSSASTTEDCIALVLKGEAD. The latter two peptides exhibited very strong cytotoxicity in MCF-7 and MDA-MB-231 cells, with 50% cytotoxic concentration values of 100 μg/mL and 100 μg/mL, respectively, in MCF-7 and 950 μg/mL and 1,000 μg/mL, respectively, in MDA-MB-231 cells (Fatemi et al., 2018). However, this study primarily employed computerized techniques to identify the active peptide fractions in human LF and preliminarily measured their anti-breast cancer activity, without investigating their anti-metastatic and invasive capabilities or the action mechanisms of these active peptides. Rahman et al. discovered that FKCRRWQWRMKKLGAPSITCVRRAF (derived from bovine LF (BLF)) exhibited a pro-apoptotic effect on breast cancer cells, leading to decreased viability in four breast cancer cells, MDA-MB-231, MDA-MB-468, SKBR3 and MCF7, as demonstrated in the MTT assay (Rahman et al., 2021). In addition, this study also found that FKCRRWQWRMKKLGAPSITCVRRAF reduced the invasion of breast cancer tumors and limited tumor growth in mice. Similar findings were obtained by Furlong et al. and Zhang et al., who stated that FKCRRWQWRMKKLGAPSITCVRRAF can promote apoptosis, increase DNA fragmentation, cause morphological changes and cause cell cycle arrest at the G2 phase in breast cancer cells. These effects were attributed to the downregulation of the mTOR signaling pathway and the upregulation of the AMPK signaling pathway (Furlong et al., 2006; Zhang et al., 2014). However, although the obtained peptides have excellent antitumor activity, their sequences are too long, and whether they can tolerate gastrointestinal digestion and be absorbed by the human body needs to be examined by *in vitro* gastrointestinal simulation or *in vivo* digestion experiments. Some short-chain anti-breast cancer peptides have also been obtained by researchers. Insuasty-Cepeda et al., Casanova et al. and Guerra et al. isolated RRWQWRMKKLG and RRWQWR from BLF, both of which led to decreased viability, increased apoptosis, and morphological changes in breast cancer cells (Casanova et al., 2017; Guerra et al., 2019; Insuasty-Cepeda et al., 2020). Additionally, the two peptides also caused mitochondrial membrane depolarization and cellular calcium overload in breast cancer cells, suggesting the activation of the mitochondria-mediated apoptosis pathway. These active peptides are relatively short in length and derived from the longer peptide FKCRRWQWRMKKLGAPSITCVRRAF, which implies that this sequence is critical for the biological activity of LF. Overall, current research on the anti-breast cancer mechanisms of LF-activated peptides is not deep enough, and more effort needs to be invested in this area in the future.

**TABLE 1:** Inhibitory effects and mechanisms of dairy-derived active peptides and peptide extracts on breast cancer progression.

Protein source	Processing method	Peptides	Model	Anti-breast cancer effects	Pathway	References
LF	Acid-pepsin hydrolysis	FKCRRWQWRMKKLGAPSITCVRRAF	MDA-MB-435 cells were treated with 25, 50 and 100 μg/mL peptide	Apoptosis, DNA fragmentation↑; morphologic characteristics changed		Furlong et al. (2006)
BLF	Solid Phase Synthesis	RRWQWRMKKLG	HTB-132 and MCF-7 were added with 50, 100, 150 and 200 μg/mL peptide	Cell viability↓; apoptosis, depolarization of the mitochondrial membrane↑; morphologic characteristics changed	Apoptotic pathway↑	Insuasty-Cepeda et al. (2020)
LF	Solid Phase Synthesis	FKCRRWQWRMKKLGAPSITCVRRAF; FK*RRWQWRMKKLGAPSIT*VRRAF; Ac-RRWQWR-NH_2_; GRRRRSVQWCA; GRRRRSVOWSA; APRKNVRWCT; APRKNVRWST; APRKNVRWCTISQPEW; GRRRRSVOWCA-P-RRWQWR-NH_2_; GRRRRSVQWCA-GG-RRWQWR-NH_2_; GIGAVLKVLTTGLPALISWIKRKRQQ-NH_2_	MDA-MB-231 were added with 10, 20 and 40 μmol/L peptide	Cell viability↓		Arias et al. (2017)
BLF	Solid Phase Synthesis	FKCRRWQWRMKKLGAPSITCVRRAF	MDA-MB-231, MDA-MB-468, SKBR3 and MCF7 were added with 100, 200, and 300 μg/mL peptide; mice was injected with MDA-MB-231-GFP-luc2 cells and treated with 4 or 5 mg peptide	Apoptosis↑; cell viability, invasion, tumor volume, tumor weight↓		Rahman et al. (2021)
BLF	Solid Phase Synthesis	RRWQWR; RRWQWRMKKLG; FKARRWQWRMKKLGA	MDA-MB-468 and MDA-MB-231 cells were added with 50, 100, 150 and 200 μg/mL peptide	Cell viability↓		Casanova et al. (2017)
BLF	Solid Phase Synthesis	RRWEWRMKKLG	MDA-MB-231 cells were treated with 1 µM peptide	Glutathione S-Transferase P1↓		Khan and Taneja (2015)
Human Lactoferrin	Solid Phase Synthesis	GEQELRKCNQWSGLSEGSVT; WSGLSEGSVTCSSASTTEDC; CSSASTTEDCIALVLKGEAD	MCF-7 and MDA-MB-231 cells were treated with 10, 100, and 1,000 μg/mL peptides	Cell viability↓		Fatemi et al. (2018)
Human milk	—	Composed of 74 amino acid residues	MCF-7 cells were treated with 60 μg/mL peptides	Cell viability↓; Apoptosis, caspase-7, DFF45↑		Semenov et al. (2010)
Human milk	Ion exchange chromatography	—	MCF-7 cells were incubated with 60 μg/mL peptide	Apoptosis, pro-caspases 8 and 9↑, mitochondrial membrane potential↓	Mitochondria mediated apoptosis pathway↑	Fomin et al. (2012)
BLF	Solid Phase Synthesis	RWQWRWQWR	MDA-MB-468 cells were added with 200, 100, 50, 25, 12.5 and 6.25 μg/mL peptides	Cell viability↓; morphological characteristics changed		Barragán-Cárdenas et al. (2020)
BLF	Solid Phase Synthesis	FKCRRWQWRMKKLGAPSITCVRRAF	T-47D, MDA-MB-231, Hs578T, and MCF-7 were added with 1.875, 3.75, 7.5, 15 and 30 µM peptide	Half maximal growth inhibitory concentration for T-47D, MDA-MB-231, Hs578T, and MCF-7 was 11.25, 21.5, 15.0, and 16.2 µM, respectively; induced cell cycle arrest at the G2 phase	mTOR signaling↓ AMPK signaling↑	Zhang et al. (2014)
BLF	Solid Phase Synthesis	RRWQWR	MCF-7 cells were treated with tetrameric peptide (15, 30 or 60 μg/mL) for 6 h	Cell viability↓; apoptosis, cellular calcium overload↑; morphologic change occurred	Mitochondria mediated apoptosis pathway↑	Guerra et al. (2019)
WPH	—	Peptide mixtures	SD rats were injected with N-methyl-N-nitrosourea	Tumor incidence, apoptotic cells, Serum C-peptide, κ-casein, BRCA1↑;		Eason et al. (2006)
Goat casein and whey protein hydrolysate	Pepsin and trypsin enzyme hydrolysis	DYRWIAL, QIMSSPWGEMYNIF, CDELGIMIWQDF, QYPYQGPIVL, SSGLGNVPRPYQL, QYKIPDWFLNR, ALPMHIR, LVMFQRR	MCF-7 cells were treated with 5, 10, 25, 50, and 100 μg/mL peptide mixtures	Cell viability, GSK 3-β, PKM2, LDH B enzymes, and MUC 1↓; apoptosis, RIPK1↑		Sahna et al. (2022)
WPH	—	Peptide mixtures	Rats were orally gavaged with DMBA	Tumor multiplicity, tumor incidence, cell proliferation, cyclin D1, IGF-2↓; Tumor latency, apoptotic cells, terminal end bud number per mm^2^, PR, β- and κ-caseins, PTEN↑	PR signaling↑; EGF signaling↓	Eason et al. (2004)
WPH	—	Peptide mixtures	MCF-7 cells were treated with various concentrations of WPH	wip1, Sesn1, Mgmt, BRCA1, Bax, P-Tp53^Ser15^, p38 MAPK, Tp53, p21↓; MCP-1↑	p38 MAPK signaling, Tp53 signaling↑	Dave et al. (2006)
Camel casein and whey protein casein and whey protein hydrolysate	Pepsin and trypsin hydrolysis	ARPKY, LIPRVKL, WNHIKRYF, FFIFTCLLAVVLAK, WSVGH, LGRVGTKCCTL, AACLLPK, FTKCKL	MCF-7 cells were treated with 400 μg/mL peptide mixtures	Cell viability↓		Taghipour et al. (2023)
Camel casein and whey protein hydrolysate	Pepsin hydrolysis	Peptide mixtures	MCF-7 cells were treated with 50–200 μg/mL peptide mixtures	Cell viability↓; ROS↑		Ibrahim et al. (2022)
Yak milk casein	Trypsin (pH 8, 37°C, 3 h) and alcalase (pH 8.0, 55°C, 3 h)	TPVVVPPFL, VAPFPEVFGK and NQFLPYPY	MCF7 and MDA-MB-231 cells were incubated with the hydrolysates (final assay dose, 0.5 mg/mL) or peptides (0, 62.5, 125, 250, 500, and 1,000 μg/mL)	Cell viability↓; apoptosis, G2/M cell cycle arrest↑	Induced G2/M cell cycle arrest	Gu et al. (2022)
Bovine colostrum whey protein hydrolysates	Pepsin-pancreatin hydrolysis	Peptide mixtures	MDA-MB-231 cells were treated with 0.03, 0.1, 0.3, 1.0, 3.0, 10.0 and 20.0 mg/mL peptide mixtures	Cytotoxicity, apoptosis↑		Fajardo-Espinoza et al. (2021)
Human αS” -casein	Chemical synthesis	YVPFP	T47D cells were added with 10^−12^, 10^−11^, 10^−10^, 10^−9^, 10^−8^, 10^−7^, and 10^−6^ mol/L peptides	Cell proliferation↓		KAMPA et al. (1996)

LF, lactoferrin; BLF, bovine lactoferricin; mTOR, mammalian target of rapamycin; DMBA, 7,12-dimethyl-benz [a]anthracene; WPH, whey protein hydrolysate.

### 2.2 Whey protein-derived anti-breast cancer peptides

Whey is a by-product of cheese manufacturing or other coagulated dairy product processing, containing 0.6% protein and 93% water, with a yellowish-green color (Zhang, 2022). Previously, whey separated from cheese production was considered a worthless by-product of the dairy industry and was difficult to process into other foods or sell as a food additive (Mehra et al., 2021). In recent years, liquid whey has gained attention for its mineral-rich, safe, easily digestible, and highly nutritious whey protein, resulting in increased effective utilization and conversion into various valuable human food supplements (Mehra et al., 2021). Whey can be converted into whey protein hydrolysate (WPH), whey protein concentrate (WPC), whey protein powder (WPP), whey protein isolate (WPI), and other metabolites using complex processing techniques including fermentation, membrane separation, chromatographic separation, and ultrafiltration (Zeng et al., 2024). These dry whey protein components consist of numerous subfractions such as β-lactoglobulin, α-lactalbumin, immunoglobulins, bovine serum albumin, proteose peptone, etc. (Zhang, 2022). In detail, WPP contains 63%–75% protein and 11.0%–14.5% lactose and its processing involves demineralization, acid, sweetening, and other reduced forms (Mehra et al., 2021). WPC has a protein content ranging from 25% to 80% and is a good source of lysine and sulfur-containing amino acids (Mehra et al., 2021; Zhang, 2022). WPI has the highest protein content (more than 90%), and generally undergoes an additional purification step to remove lactose and fat, making it popular among fitness enthusiasts (Chen et al., 2023). Interestingly, when WPP, WPC, and WPI are treated with acids, enzymes, or heat, the intact protein breaks down into polypeptides, smaller peptides, and amino acids, resulting in WPH (Mehra et al., 2021). Various whey proteins and WPH provide high-quality protein that can enhance athletic performance without toxic effects (Mangano et al., 2019; Zhao and Ashaolu, 2020). Specifically, whey protein, whey protein-derived peptides, and WPH are considered to have no adverse effects on body weight, food intake, behavior, organ weight, activity levels, or blood and urine parameters. They do not lead to abnormal changes in overall health, biochemical indicators, histopathological findings, or other observational metrics, nor do they exhibit acute or subacute toxicity (Zhao and Ashaolu, 2020). However, some studies suggest that excessive intake of certain bioactive peptides from whey protein may cause adverse reactions or allergies, though these anomalies can be mitigated through extensive hydrolysis (Cui et al., 2023). At present, scholars have isolated a large number of peptides from WPH, such as antioxidant peptides, anti-cancer peptides, ACE inhibitory peptides, anti-diabetic peptides, and immune-enhancing peptides (Zhao et al., 2022). These peptides typically exhibit stability to pH, ions, and heat, but their integrity may be compromised when exposed to the gastrointestinal environment (Adjonu et al., 2023; Wu et al., 2024). Importantly, research indicates that bioactive peptides maintain their biological activity, even when fully hydrolyzed into smaller peptide fragments (Tavares et al., 2011; Jiang et al., 2018).

Currently, the anti-breast cancer effects of whey protein-derived active peptides have received widespread attention. Various bioactive peptides, such as DYRWIAL, ARPKY, LIPRVKL, ALPMHIR, and LVMFQRR, have been isolated from fermented or enzymatically hydrolyzed whey proteins, demonstrating antitumor activity in breast cancer cells and/or animal models (Sahna et al., 2022; Taghipour et al., 2023). Except for individual active peptides, researchers have also investigated the anti-breast cancer effects of peptide mixtures from whey proteins of camel, goat, and cow (Dave et al., 2006; Sahna et al., 2022; Taghipour et al., 2023). Anti-breast carcinogenesis peptides and hybrid peptides identified from the aforementioned animal-derived whey proteins have shown effectiveness in reducing cancer cell proliferation, adhesion, and migration, promoting free radical accumulation, inducing apoptosis in cancer cells, and leading to the excessive expression of apoptosis-related proteins (Eason et al., 2004). Furthermore, investigations have reported that these peptides act mainly by activating several signaling pathways such as p38 MAPK and p53 signaling (Figure 2) (Dave et al., 2006).

Sahna et al. used pepsin and trypsin enzymes to hydrolysate goat casein and goat whey protein and obtained eight anti-cancer peptides consisting of 7–14 amino acids (Sahna et al., 2022). The research showed that the IC_50_ values (the concentration of the peptide required to achieve 50% viability in MCF-7 cells) of pepsin-treated whey protein hydrolysate, trypsin-treated whey protein hydrolysate, pepsin-treated casein hydrolysate, and trypsin-treated casein hydrolysate were 33.38, 23.49, 34.83 and 34.23 μg/mL, respectively. Besides, these peptide fragments also downregulated the expression of GSK 3-β, PKM2, LDH B, and MUC 1, while regulating RIPK1 levels, which may reveal the anticancer mechanism of these active peptides. Whey protein hydrolyzed peptides are the most studied active peptides, and anticancer peptides have also been isolated from them for research purposes. According to Dave et al. and Eason et al., the whey protein hydrolysate exhibited anti-tumor effects in MCF-7 cell and rat models, as evidenced by a decrease in tumor diversity, tumor incidence, and cell proliferation, as well as reduction in the expression of cyclin D1 and IGF-2 (Eason et al., 2004; Dave et al., 2006). There was also an upregulation of apoptotic cell proportions and terminal end bud number per mm^2^, along with the activation of some important anticancer factors (MCP-1, PTEN, PR) and inhibitory activation of oncogenic factors (p21, wip1, Sesn1, Mgmt, BRCA1, Bax), which may be attributed to the activation of multiple oncogenic signaling pathways such as p38 MAPK, Tp53, and PR signaling. However, neither study identified the specific anticancer peptide sequences contained in whey protein hydrolysates. Therefore, future studies could attempt to categorize the active whey protein-derived peptides into different fractions according to molecular weight and identify the most potent biopeptides with anti-breast cancer activity from these mixtures. In addition, probiotic fermentation presents a promising way to obtain active peptides from whey proteins, an area that has yet to be extensively explored.

### 2.3 Casein-derived anti-breast cancer peptides

Casein is a type of insoluble phosphoprotein and constitutes 80% of the milk proteins (Wusigale et al., 2020; Auestad and Layman, 2021). It is one of the predominant proteins in milk and can be isolated by precipitating milk at pH 4.6, as well as through techniques such as chromatography, electrophoresis, enzymatic treatment, and membrane filtration (Wusigale et al., 2020). Structurally, casein consists of four subfractions, αS1-, αS2-, β-, and κ-caseins, which account for approximately 38%, 10%, 36%, and 13% of the casein fraction, respectively (Auestad and Layman, 2021). These four casein subfractions are amphiphilic, with molecular weights ranging from 19 to 25 kDa and average isoelectric points between 4.1 and 5.3. This unique structure allows them to readily self-assemble into stable micellar structures in aqueous solutions, which can be destroyed in the presence of rennet (Wusigale et al., 2020). Casein is considered safe and non-toxic, with no mutagenic or clastogenic effects, and does not cause developmental, reproductive, or target organ toxicity (Dent et al., 2007; Phelan et al., 2009). Research by Dent et al., Doorten et al. and Phelan et al. robustly demonstrated that casein hydrolysates or their derived peptides, at doses not exceeding 2 g/kg body weight per day, did not induce any adverse reactions, nor did they cause abnormal changes in the eyes, nervous system, urine, blood, or kidneys (Dent et al., 2007; Doorten et al., 2009; Phelan et al., 2009). More importantly, casein provides the body with a well-balanced profile of essential amino acids, characterized by extremely high levels of proline (accounting for 16% of the total amino acids) and nearly complete absence of cysteine. This results in a structure that lacks the disulfide bridges and alpha-helices typically found in most proteins (Auestad and Layman, 2021). The biological activity of casein has also been widely reported by scholars. Some bioactive peptides have already been identified from casein by enzymatic hydrolysis methods, including alcalase, fermentation, and chemical and physical hydrolysis, and these peptides exhibit stability against heat, acidic conditions, and gastrointestinal enzymes (Quirós et al., 2009; Contreras et al., 2013; Wu et al., 2014; Kumar et al., 2016; Xue et al., 2021). Some of these bioactive peptides exhibit immunomodulatory, antioxidant, gut regulatory, and nutrient-absorption promoting effects locally in the intestinal cavity, while others need to be absorbed into the bloodstream to exhibit bioactivities. Therefore, studying both intact peptide absorption and peptide bioactivity is equally significant.

Anticancer peptides in casein proteins have been widely investigated, such as anti-ovarian cancer peptide PGPIPN, anti-melanoma peptide INKKI, and anti-leukemia peptide FFSDK (Sah et al., 2015). However, research on casein-derived peptides specifically targeting breast cancer has been limited, with only a few studies focusing on yak milk casein and human αS”-casein reporting the activities of anti-breast cancer peptides. For example, Gu et al. isolated three anti-breast cancer peptides, TPVVVPPFL, VAPFPEVFGK and NQFLPYPY, from yak milk casein and reported that these peptides exhibited excellent anticancer effects in both MCF-7 and MDA-MB-231 breast cancer cell models (Gu et al., 2022). These effects were characterized by an apparent increase in cancer cell death, a significant reduction in cell viability and cell cycle arrest at G2/M phase. Another study by KAMPA et al. also stated that YVPFP, an anti-breast cancer peptide derived from human αS”-casein, significantly (*p* < 0.05) decreased the proliferation of T47D cells (Kampa et al., 1996). Unfortunately, there is insufficient research on casein-derived anti-breast cancer peptides to draw definitive conclusions. Future efforts could focus on identifying active peptides from the four casein subfractions and comparing their anticancer activities in experimental models. In addition, while most of the identified active peptides or peptide mixtures have validated their anticancer activity in cancer cell models, animal transplantation tumor models are also very important for confirming experimental results or exploring anti-breast cancer mechanisms.

## 3 Anti-breast cancer activities of dairy products

Some scholars have also studied the anti-breast cancer activity of dairy products, with some popular examples including fermented milk, kefir, and cheese (Table 2). These fermented dairy products often contain a large number of anti-breast cancer active peptides and other anticancer bacterial metabolites. Identifying these anticancer active ingredients and investigating the anticancer effects and mechanisms of dairy products have become prominent areas of research in functional foods.

**TABLE 2:** Inhibitory effects and mechanisms of fermented or non-fermented dairy products on breast cancer progression.

Dairy products	Processing method	Model	Anti-breast cancer effects	Others	References
Fermented milk	Three *Bifidobacterium* (*B. infantis* Bb-02, *B. bifidum* Bb-11, and *B. animalis* Bb-12) and two *Lactobacillus* (*L. acidophilus* La-05 and *L. paracasei* subsp. *paracasei* Lc-01)	MCF-7 cells were added with fermented milk	Cell growth↓	pH: 3.91–4.72	Biffi et al. (1997)
Fermented milk	*L. helveticus* R389 or *L. helveticus* L89	BALB/c mice were injected with 4T1 cells and were fed with fermented milk	Tumor volume, IL-6↓, TNF-α, IL-4, IL-10, IFNγ, apoptosis↑	Immune enhancement	de LeBlanc et al. (2005a)
Fermented milk	*L. helveticus* R389 or *L. helveticus* L89	BALB/c mice were injected with 4T1 cells and were fed with fermented milk	IFNγ, IL-6↓, TNF-α, IL-4, IL-10, CD4^+^ cells↑	Immune enhancement	de LeBlanc et al. (2005b)
Fermented milk	*L. helveticus* R389	BALB/c mice were injected with 4T1 cells and were fed with fermented milk	Tumor volume, lymphocytes and monocytes infiltration, TNFα, INFγ, IL-10 and IL-6↓; leucocytes and neutrophils numbers, CD4^+^ and CD8^+^ T cells, apoptotic cells, postinoculation of tumor cells↑	β-glucuronidase values↓	Rachid et al. (2006)
Fermented milk	*Lactobacillus casei* ATCC 393	MCF-7 cells were added with 25–50 μL WSE	Antiproliferative activity↑	DKIHPF, VVPPFLQPE, LLYQEPVLGPVRGPFPIIV, LLYQEPVLGPVRGPFPII, ELQDKIHPF, LYQEPVLGPVRGPFPIIV, LHLPLPLLQSW, NLHLPLPLLQSW, DVENLHLPLPLLQSW, LYQEPVLGPVRGPFP, MPFPKYPVEPF, YQEPVLGPVRGPFPIIV, LLYQEPVLGPVRGPFP, NLHLPLPLL, NENLLRFF, NLLRFF, FVAPFPEVFGKE, ENLLRFF, RFFVAPFPEVFGKE, ALINNQFLPYPYYAKPA, KYIPIQYVL	Abdel-Hamid et al. (2019)
Fermented milk added with fresh royal jelly	*Lactobacillus helveticus* Lh-B02	MCF-7 cells were treated with 200 μL of fermented milk samples containing different concentrations of fresh royal jelly (0.5, 1% and 1.5%)	IC50 decreased from 48.7 μg/mL to 32.15, 13.97 and 9.86 μg/mL, respectively	Viscosity: 640.8–648.7 cP; water holding capacity: 58.3%–62.88%; DPPH: 62.28%–73.47%; ABTS: 30.15%–58.36%; Overall acceptability on day 0: 97.00	Hassan et al. (2022)
Camel milk	*Lb. plantarum* DSM2648	MCF-7 cells were treated with 25 µL of filtered WSE	Proliferation inhibition↑	ABTS, DPPH, α-Amylase and α-glucosidase inhibition, ACE inhibition↑	Ayyash et al. (2018)
Yogurt added with *Portulaca oleracea* whole plant	*Lactobacillus acidophilus*, *Bifidobacterium lactis*, and *Streptococcus thermophilus*	MCF-7 cells were added with 50 and 350 μg/mL *Portulaca oleracea* whole plant	Cell viability↓	pH: 4.50, acidity: 81.33 mg/10 mL, fat: 1.45%, viscosity: 198.2 cP; water holding capacity: 7.7 mL/g; total phenolic content, overall acceptability, lactic acid bacteria count↑; MDA↓;	Al-Quwaie et al. (2023)
Yogurt produced by reconstituted skim milk powder	*Bifidobacterium animalis subsp. lactis* BB-12 and *Lactobacillus acidophilus* ATCCSD 5221	MDA-MD231 and SKBR3 cells were added with 1/6, 1/5, 1/4, 1/3, and 1/2 concentrations	Cell viability↓	mean pH drop rate: 0.0055–0.0060; mean acidity increase rate: 0.20–0.25 ◦D; mean redox potential increase rate:0.33–0.37 mV	(Parvarei and Mortazavian)
Kefir	Kefir grains	Mice were injected with 4T1 cells and administrated for kefir for 7 consecutive days	Tumor growth, tumor volume↓; CD4^+^ T Lymphocytes, apoptosis↑	Immune enhancement	de LeBlanc et al. (2007)
Kefir	Kefir grains (2%–10%)	MCF-7 cells were added with 0.31%, 0.63%, 1.25%, 2.5%, 5.0% and 10.0% vol/vol kefir extracts	Cell proliferation↓	—	Chen et al. (2007)
Kefir	Kefir grains	Mice were injected with 4T1 cells and administrated with kefir for 7 consecutive days	Tumor volume, IFNγ, IL-6↓; TNF-α, IL-10, IL-4, T-helper CD4 cells, CD8 T cells↑	Immune enhancement	de LeBlanc et al. (2006)
Kefir	Kefir grains	Mice were injected with 4T1 cells and administrated with kefir at a concentration of 150 mg/kg body weight per day	Cell cytotoxicity, apoptosis, IFN-γ and IL-2↑; cell migration, invasion, tumor volume, tumor weight, mitosis, metastasis, IL-10 and IL-1β, lipid peroxidation and NO Levels↓; ICAM, iNOS, MMP-9, NF-κB, G-CSF, GM-CSF, IL-4, TNF-α, iNOS↓; *ex vivo* angiogenesis was obstructed	CD4 cells, CD8 cells↑	Zamberi et al. (2016)
Kefir	*L. kefiri* P-B1	MCF-7 cells were treated with 0.6, 1.25, 2.5 and 5.0 mg/mL peptides; Female Swiss albino mice were injected with breast cancer cells and fed with kefir at a dose of 2 g/kg/day	Cell survival↓; Tumor growth, tumor weight, cell proliferation, MMP, Bcl2↓; apoptosis, p53, p21, p27, bax, caspase3, CD4^+^ T and CD8^+^ T cells, lymphocytes, TNF-α↑; cell cycle arrest at G0/G1 phase	Induced G0/G1 cell cycle arrest; p53 signaling pathway↑; immune enhancement	El-Din et al. (2020)
Kefir	Kefir grains (2%–10%)	MCF-7 cells were treated with 0.31%, 0.63%, 1.25%, 2.50% and 5.0% (v/v) kefir mother culture extracts	Cell proliferation↓		Chen et al. (2011)
Cow and ewe cheeses made with saffron	Cheese ripens from 15 days to 22 months	MDA-MB-231 cells were treated with crocin-rich extracts from cheese	Cell viability↓	Total crocins: 0.54–30.57 mg/100 g	Ritota et al. (2022)
Enzyme-modified cheese	Neutrase™,Flavorpro™ 937MDP, Lipomod™ 801MDP	MCF-7 cells were treated with 0.0186, 0.0556, 0.1667 and 0.5 mg/mL of cheese WSE	Cell viability↓	Peptide content: 14.4–231.5 mg tryptone/g DM; 678.5–764.5 mg tryptone/g DM	Akilli et al. (2023)
Himalayan cheese	*Lactobacillus plantarum* (NCDC 012), *Lactobacillus casei* (NCDC 297), and *Lactobacillus brevis* (NCDC 021)	MCF-7 cells were added with 50 μL of different cheese WSE	Cell viability↓	Cheese matrix is more compact with proteins and low number of fat globules	Mushtaq et al. (2019)
Low-fat Akawi cheese made from blends of bovine and camel milk	*Streptococcus thermophilus* and *Lactobacillus bulgaricus*	MCF-7 cells were added with 25 µL of filtered cheese WSE	Cell proliferation↓	pH↓; lactic acid bacteria count, α-Amylase and α-glucosidase inhibition; ACE inhibition↑	Ayyash et al. (2021)

bLf, bovine lactoferrin; GSH, glutathione; Se-β-Lg, seleno-β-lactoglobulin; MMP, mitochondrial membrane potential; DM, dry matter; WSE, water soluble peptide extract; DPPH, 2,2-diphenyl-1-picrylhydrazyl radical scavenging activity; ABTS, 2,2′-azino-bis(3-ethylbenzothiazoline-6-sulfonic acid scavenging activity.

### 3.1 Anti-breast cancer effects of fermented milk

The demand for fermented milk has been rapidly increasing due to the growing vegetarian population and the rising popularity of high-protein diets (Sakandar and Zhang, 2021). The global fermented milk market was valued at USD 320.60 billion in 2022 and is expected to grow at a CAGR of 4.9% from 2022 to 2031, potentially reaching nearly $500 billion by 2031 according to Straits Research. Fermented milk is produced through the fermentation and acidification of milk by live microorganisms, typically *Lactobacillus bulgaricus* and *Streptococcus thermophilus*, resulting in the breakdown and utilization of macromolecules in the milk, and further the production of peptides, bacteriocins, free fatty acids, and conjugated linoleic acid, finally enhancing bioavailability and thickness of the products, increasing the number of viable bacteria, and prolonging their shelf life (Alhaj and Kanekanian, 2014; Hadjimbei et al., 2022). Traditionally, milk from various mammalian species, including cows, sheep, goats, camels, mares, buffaloes, and yaks, can be used to prepare fermented milk products. However, the flavor, composition, and nutritional value of the final product are affected by factors such as geographic and climatic conditions, animal health, species, feeds, seasons and stages of lactation, strains of fermenting bacteria, and fermentation conditions (Bintsis and Papademas, 2022). Commonly used milk starters are *Lactococcus lactis subsp. cremoris*, *Str. Thermophilus*, *Lc. lactis subsp. lactis*, *Lb. delbrueckii* subsp*. delbrueckii*, *Lb. delbrueckii subsp. lactis*, *Lb. helveticus*, *Leuconostoc spp*., etc. (Bintsis and Papademas, 2022). They are safe and beneficial for enriching the natural flora of the human gut. Additionally, fermented milk is a good source of calcium, phosphorus, potassium, vitamin A, vitamin B2, vitamin B12, high biological value proteins and essential fatty acids (Ilesanmi-Oyelere and Kruger, 2020). They also offers unique flavors, smooth textures, appealing appearances, enhanced digestibility and specific benefits such as anti-fatigue, antioxidant, anti-inflammatory, anti-cancer, intestinal modulation, cardiovascular disease improvement, serum cholesterol-lowering effects, etc., as compared to regular milk, which makes them popular among consumers and researchers (HoSoNo et al., 2002; Szajnar et al., 2020; Abdelrazik and Elshaghabee, 2021; Zhang D. J. et al., 2023; Zhang Z. Q. et al., 2023).

Due to the existence of various anticancer components, such as active peptides, probiotics, short-chain fatty acids, and probiotic metabolites, the effect of fermented milk on cancers has been widely studied. Some scholars have focused on identifying anticancer peptides from fermented milk. Abdel-Hamid et al. used *Lactobacillus casei* ATCC 393 to prepare fermented milk and characterized the composition of the obtained products (Abdel-Hamid et al., 2019). They found that the fermented milk contained a bacterial population exceeding 9 log cfu/g, with a pH value of 4.52, titratable acid content of 0.73%, and proteolytic activity of 0.36. Additionally, both the water extract of fermented milk (WSE) and the fermented milk extract with a molecular weight of less than 2 kDa (F1) exhibited ACE-inhibitory, antioxidant, and anti-breast cancer activities. Notably, the antiproliferative activity of the F1 fraction was 64.25% on day 0 and this value increased to 78.90% on days 21 of storage. Finally, they also identified the sequences of active peptides in F1 and successfully obtained several peptides such as DKIHPF, VVPPFLQPE, ELQDKIHPF, LHLPLPLLQSW, NLHLPLPLLQSW, LYQEPVLGPVRGPFP. However, a limitation of this study is that no specific anti-breast cancer studies were conducted on the identified active peptides. Biffi et al., de LeBlanc et al., de LeBlanc et al. and Rachid et al. fermented cow’s milk with different starter bacteria and found that the extracts inhibited breast cancer progression in mouse and cellular models (Biffi et al., 1997; de LeBlanc et al., 2005a; de LeBlanc et al., 2005b; Rachid et al., 2006). Specifically, fermented milk inhibited breast cancer cell proliferation, reduced tumor size, and modulated cytokines (downregulated IL-6 levels and upregulated TNF-α, IL-4, and IL-10 levels). In addition, fermented milk enhanced the immune function of BALB/c mice, as evidenced by an increase in the number of CD4^+^ and CD8^+^ T cells. In recent years, the addition of other natural anticancer-active ingredients into fermented milk has become a hot topic. Hassan et al. discovered that incorporating fresh royal jelly into *Lactobacillus helveticus* Lh-B02 fermented milk significantly increased its anti-breast cancer activity, with the IC_50_ value of the fermented milk extract decreasing from 32.15 μg/mL to 9.89 μg/mL as the royal jelly addition concentration increased from 0.5% to 1.5% (Hassan et al., 2022). The addition of natural components, especially polyphenols, significantly improves the anticancer activities of fermented milk, although it may also alter other properties, such as color, viscosity, texture and sensory scores. Thus, these indicators should be taken into account when preparing fermented milk with added anticancer components. Some scholars have also explored the anti-breast cancer effects of fermented camel milk. On the first day of fermentation, fermented camel milk showed lower antiproliferative activity (41.5%) against MCF-7 cells compared to fermented cow’s milk (44.9%), however, after 21 days of post-ripening, the antiproliferative activity of fermented camel milk (47.9% (fermented camel milk) vs. 44.8% (fermented cow’s milk)) was significantly enhanced. Therefore, camel milk might be a better anticancer food ingredient than cow’s milk, and future research on fermented camel milk could focus on identifying anti-breast cancer peptides, and exploring the *in vivo* anticancer effects and anticancer mechanisms. In addition, the anti-breast cancer activity of dairy products such as fermented sheep, goats, mare, buffalo, and yak milk is also worth exploring.

Researchers have also explored the beneficial effects of fermented milk as an immunoadjuvant in breast cancer chemotherapy. In one study, milk fermented by *Lactobacillus casei* CRL431 not only enhanced the therapeutic efficacy of capecitabine but also effectively prevented cancer cell metastasis and alleviated the toxicity associated with capecitabine treatment (Utz et al., 2021). This was evidenced by reduced weight loss in mice, restored leukocyte counts and hematocrit levels, decreased diarrhea scores, improved inflammatory markers (IL-6, TNF-α, IL-10, IFN-γ, and MCP-1), and a higher villi length to crypt depth ratio. Overall, fermented dairy products are most likely to be initially applied as adjunctive agents in the clinical treatment of breast cancer.

### 3.2 Anti-breast cancer effects of yogurt

Yogurt is one of the most widely consumed and oldest fermented dairy products. It is prepared by fermenting and acidifying milk with bacterial culture (primarily *Lactobacillus delbrueckii* subsp. *bulgaricus* and *Streptococcus thermophilus*, but some products also use *Bifidobacterium* species, *Lactobacillus helveticus*, etc.), resulting in a thickened, more easily digestible product with an attractive flavor and a longer shelf life (Olson and Aryana, 2022; Hasegawa and Bolling, 2023). Yogurt is typically made from the milk of cows, buffalo, sheep, goats, and other livestock, and it can be categorized into various types, including plain yogurt, whipped yogurt, fruit-flavored yogurt, Greek yogurt, and frozen yogurt (Aryana and Olson, 2017; Oz et al., 2023). According to Statista, yogurt sales in the U.S. reached $7.24 billion in 2021, compared to $5.58 billion in 2011, implying a robust growth rate in the yogurt market over the past decade (Olson and Aryana, 2022). Nutritionally, yogurt provides highly digestible and bioavailable proteins, calcium, potassium, vitamin A and vitamin B_12_, and its rich components of live microorganisms and their metabolites also serves to nourish the intestinal tract (Webb et al., 2014). From a flavor perspective, more than 90 different volatile compounds have been detected in yogurt, including acids, alcohols, aldehydes, ketones, esters, etc. Key contributors to the desirable flavor of yogurt include lactic acid, acetoin, acetaldehyde, diacetyl, and 2-butanone (Chen et al., 2017).

Yogurt hydrolysates are rich in bioactive peptides and free amino acids, making them important resources for cancer prevention (Çakmakoglu et al., 2024). Kefir is one of the best-known branches of yogurt, with origins tracing back to the Balkans, Eastern Europe and the Caucasus, but nowadays its consumption has expanded to other parts of the world due to its excellent health-promoting effects (Azizi et al., 2021). Kefir is also an acidic and viscous fermented beverage prepared by fermentation carbonation of kefir grains with milk or water, typically composed of 89%–90% water, 6.0% sugar, 3.0% protein, 0.2% lipids, 0.7% ash, and 1.0% lactic acid and alcohol (Azizi et al., 2021). The main fermenting bacteria in kefir are lactic acid bacteria and yeasts, with the former mainly comprising *Lactobacilli*, *Lactococci*, *Streptococci*, etc., while the latter primarily containing *Candida* sp., *Kluyveromyces* sp., *Saccharomyces* sp., etc. (Arslan, 2015). The symbiotic metabolism of these complex mixtures contributes to protein hydrolysis and lipolytic degradation, thereby imparting a unique flavor to kefir (mainly derived from lactic-, acetic-, hippuric-, butyric-, pyruvic-, propionic-acid, diacetyl and acetaldehyde) (Arslan, 2015). However, due to the complexity of kefir colonies, the biological activity of the obtained kefir products varies largely, sparking interest among researchers in optimizing fermentation conditions and ingredients to enhance the functional properties of fermented dairy products.

The anti-breast cancer effects of kefir have been revealed in several studies. Scholars have stated that kefir reduces tumor cell viability and growth rates, regulates cytokine expression in tumor tissues (e.g., downregulates IL-6, IL-10 and IL-1β, upregulates TNF-α, IL-10, and IL-4), promotes apoptosis, and exerts immune-enhancing effects. As reported by Zamberi et al., kefir inhibited the growth and metastasis of breast cancer in both *in vitro* and *in vivo* experiments (Zamberi et al., 2016). In addition, kefir reduced lipid peroxidation and NO levels *in vivo*, attenuated the accumulation of inflammatory factors of IL-4, TNF-α, iNOS, IL-10, and IL-1β, enhanced the occurrence of blocked angiogenesis as well as induced a 5-fold increase in CD4^+^ T levels and a 7-fold increase in CD8^+^ T cells. These findings were corroborated by studies conducted by de LeBlanc et al. (de LeBlanc et al., 2006; de LeBlanc et al., 2007). The anticancer effects of kefir may stem from its ability to activate a series of apoptotic pathways (Bcl2↓, bax, caspase3↑) and tumor signaling inhibitory pathways (p53, p21, p27↑), as well as to induce immune enhancement, such as upregulation of CD4^+^ T and CD8^+^ T cells (El-Din et al., 2020). However, there are few studies that simultaneously focus on the quality, anticancer effects and anticancer components of kefir.

In addition to kefir, plain yogurt also exhibits anti-breast cancer activity in several studies. Parvarei and Mortazavian found that yogurt fermented with *Bifidobacterium animalis subsp. lactis* BB-12 and *Lactobacillus acidophilus* ATCCSD 5221 significantly reduced the activity of MDA-MD- 231 cells (Parvarei and Mortazavian, 2022). However, this study did not measure other anti-cancer indicators. A more popular research direction involves the addition of natural anticancer substances to yogurt. For example, Al-Quwaie et al. incorporated *Portulaca oleracea* extract into yogurt and evaluated its fat (1.45%), pH (4.50), titratable acid (81.33 mg/10 mL), viscosity (198.2 cP), and water holding capacity (7.7 mL/g) (Al-Quwaie et al., 2023). The addition of *Portulaca oleracea* not only improved the acceptability and *Lactobacilli* content of yogurt but also enhanced its anticancer potential and effectively inhibited the accumulation of malondialdehyde (MDA). Further investigations are needed to explore the effects of adding various probiotics or natural actives to yogurt to further enhance its anticancer efficacy.

### 3.3 Anti-breast cancer effects of cheese

Cheese is one of the most ancient dairy-based fermented products that was first produced in various Western Asia countries around 8,000 years ago. It is consumed by a huge amount of the world’s population, regardless of age or origin (Zheng et al., 2021; Zhang et al., 2024a). Recently, cheese consumption has been growing at an average annual rate of 3%, with global sales reaching $122.1 billion due to increased demand for healthier, more nutritious, and more convenient foods (Hutchins and Hurley, 2024; Zhang et al., 2024a). About 2,000 types of cheese are reported, with well-known varieties including Cheddar, Mozzarella, and Parmesan (Feeney et al., 2021). These cheeses are generally made from natural ingredients, such as fresh milk (cows, buffaloes, camels, goats, sheep and donkeys), starter culture, salt and enzymes, through a series of fermentation processes (Ozcan and Kurdal, 2012; Feeney et al., 2021; Bittante et al., 2022). Most cheeses are prepared by coagulating milk with rennet and then ripening for about 2 weeks to 2 years. Other cheeses are manufactured via acid coagulation or a combination of heat and acid and are typically consumed fresh (Feeney et al., 2021). From a nutritional standpoint, cheese can be recognized as a promising macronutrient-rich food, with a high fat and protein content, as well as a micronutrient-rich food, with considerable concentrations of vitamins A, B_2_ and B_12_ and calcium (Zhang et al., 2024a). The ultimate consumer acceptance of cheese largely depends on its sensory characteristics, such as flavor, texture and aroma, which are determined by the various compounds and molecules that constitute it, including fatty acids, ketones, amines, free amino acids, active peptides, alcohols and aldehydes (Zheng et al., 2021).

The frenzy of cheese consumption has also prompted researchers to investigate its potential health benefits. It is well established that cheese has anti-hypertensive, antifungal, antibacterial, antioxidant, and gut-regulating properties. Thus, isolating highly active substances from cheese, including anticancer peptides, is of high value and can promote the development of functional foods (Sprong et al., 2010; Bernabucci et al., 2014; Théolier et al., 2014; Chen et al., 2019). According to Akilli et al., the active peptide content in digested and undigested enzyme-modified cheese was found to be 678.5–764.5 mg tryptone/g DM (dry matter) and 14.4–231.5 mg tryptone/g DM, respectively (Akilli et al., 2023). These active peptides exhibited anti-breast proliferative effects against MCF-7 cells. In addition, the anticancer activity of cheese became higher with the extension of storage time, likely due to an increase in the proportion of small peptides resulting from protein hydrolysis during ripening, thus enhancing cancer cytotoxicity. The study of Ayyash et al. found that the anti-proliferation activity of cheese against MCF-7 cells (about 55%) reached the highest at camel milk to bovine milk ratio of 30:70 (Ayyash et al., 2021). After 30 days of storage, the anti-proliferation activity of cheese further increased to ∼90%. However, this study did not explore the effects of ripening time on the active components of cheeses, leaving the specific reasons for the enhanced anticancer activity unclear. The effect of different starter cultures on the anticancer activity of cheese was also investigated. Cheeses fermented with *Lactobacillus plantarum* NCDC 012, *Lactobacillus casei* NCDC 297, or *Lactobacillus brevis* NCDC 021 presented a significant increase in anticancer activity compared to cheeses without starter bacteria. However, no significant differences in proliferation inhibition were observed between different cheese extracts. The enhanced anticancer activity could be due to the massive release of bioactive peptides triggered by the starter culture, thus initiating a cascade of caspase cleavage, or it might be attributed to the competitive binding of peptides and cancer growth factors to cancer cell membrane receptors, leading to apoptosis of cancer cells.

Several active or functional ingredients can be added to cheese for a variety of reasons, such as enhancing flavor, increasing bioactivity and therapeutic effects, and improving texture and sensory scores. There are few studies focused on incorporating anticancer ingredients into cheese. In one notable study, saffron, a traditional Chinese medicine known for its antidiabetic, antithrombotic, anti-depression, anti-anxiety, and insomnia-relieving effects, was added to cheese curd. The obtained cheeses exhibited higher anti-breast cancer activity than cheeses made by adding saffron directly to cheese milk. In addition, stronger anticancer effect was observed after 19 months of cheese ripening, with the cheese extract reducing the activity of MDA-MB-231 cells to less than 60%. This study lays the foundation for supplementing other anticancer ingredients into cheese. Polysaccharides and protein hydrolysates have been reported to improve cheese quality as well as inhibit the growth of breast cancer cells making them potential candidates for blending into cheese milk in the future (Taniya et al., 2020; Wan et al., 2020; Zhang D. J. et al., 2021; Zhang et al., 2024b).

## 4 Anti-breast cancer activities of dairy protein-based nanoparticles

Nanotechnology is a crucial cutting-edge technology that facilitates contribution, development and sustainable impacts in all sectors of human activity, involving food, medicine and agriculture, and provides innovative applications and useful solutions for related industries (Wasilewska et al., 2023). Nanoparticles are synthetic particles with sizes ranging from 1 to 1,000 nm, serving as intermediate structures between atoms and microscopic objects (Wasilewska et al., 2023). Their appearance can be spherical, conical, tubular, cylindrical, hollow, helical, etc., or irregular, with significant variability in size, structures and crystalline morphology (Joudeh and Linke, 2022). In terms of material, nanoparticles can be categorized into three main categories: organic, carbon-based, and inorganic. Organic nanoparticles are further divided into carbohydrate-based, protein-based, lipid-based, and polymer-based nanoparticles, which have the advantage of being naturally nontoxic, biodegradable, and partially cavity-containing. Carbon-based nanoparticles includes fullerenes, carbon black nanoparticles, and carbon quantum dots, etc., which are characterized by high strength, electrical conductivity, special optical and thermal characteristics as well as excellent biocompatibility (Joudeh and Linke, 2022). Inorganic nanoparticles are mainly metal-based, ceramic-based and semiconductor-based nanoparticles, which demonstrate high stability and excellent loading capacity compared to other nanoparticles (Joudeh and Linke, 2022). In the cross fields of food and medicine, nanoparticles can be used to deliver bioactive compounds to the target organs or tissues, which has greatly advanced the application of nanoparticles in the food, nutraceutical, and pharmaceutical industries (Wen et al., 2022; Luo et al., 2023). These technologies not only protect active substances such as active peptides, polyphenols, vitamins, minerals, essential fatty acids, flavors, and antioxidants, but also control the release speed of drugs or bioactive compounds, ensuring their precise delivery and greatly improving therapeutic efficacy against various diseases (Luo et al., 2023; Zhang Z. Q. et al., 2023). Specifically, nanoparticles prevent damage to the active substance caused by the harsh environment of the gastrointestinal tract, improve drug absorption/permeation in the gastrointestinal tract, and target drugs or active substances to specific cells, tissues, or organs in the human body through passive or active transport pathways (Martins et al., 2023).

Dairy protein-based nanoparticles are considered safe, efficient, highly loaded and easy to prepare (Tavakoli et al., 2021). The main types of nanoparticles using dairy protein as wall material contain β-lactoglobulin nanoparticles, LF nanoparticles, whey protein nanoparticles and casein nanoparticles (Table 3). These nanoparticles offer advantages such as low cytotoxicity, excellent biocompatibility and biodegradability, and enhanced specific delivery (Tavakoli et al., 2021). In addition, several studies have utilized dairy protein hydrolysates or dairy protein-derived active peptides as encapsulated substances within nanoparticles, which effectively prevents the inactivation of active peptides in the hostile environment of moisture, gastric acid, and digestive enzymes, all of which ensures the precise delivery of the drug to the intestinal tract (Zhang Z. Q. et al., 2023). Both scenarios have been applied in the treatment of breast cancer, typically exhibiting more effective breast cancer cell-killing effects and significantly inhibiting the invasion, migration and metastasis of cancer cells, eventually leading to better therapeutic effects (Abu-Serie and El-Fakharany, 2017; Atallah et al., 2022). LF nanoparticles are currently one of the most studied nanoparticles, featuring unique advantages such as small particle size, injectability, excellent bioactivity and certain loading capacity (Tavakoli et al., 2021). The combination of LF and polysaccharides to prepare nanoparticles for the treatment of breast cancer is a hot research direction. Popular examples such as LF-chitosan, LF-pectin, LF-polysaccharide carboxy methyl cellulose, etc., have garnered substantial attention as prospective materials (Aval et al., 2021; Abu-Serie and El-Fakharany, 2017). The bovine lactoperoxidase (LPO), LF and chitosan nanoparticles (NPs) were crosslinked by Abu-Serie and El-Fakharany to obtain LF coat LPO-loaded NPs (Abu-Serie and El-Fakharany, 2017). The loading capacity, encapsulation efficiency (EE), particle size and zeta potential of these prepared NPs were 62.3%, 88.7%, 477.5 nm and 20.6 mV, respectively. Besides, LF coat LPO-loaded NPs inhibited cancer cell viability (IC_50:_ 150.1 ± 4.8 μg/mL), induced apoptosis (apoptotic cells: 47%), and blocked the cell cycle, which was attributed to the regulation of NF-κB, Bcl-2 and p53 by the NPs. However, this study only explored the *in vitro* anticancer effects of the NPs, leaving unanswered whether they can be effectively released and exert similar efficacy *in vivo*. Considering LF is a protein with a high affinity for trivalent iron, some researchers and scholars have also explored the anticancer effect of LF nanoparticles saturated with trivalent iron ions. Kanwar et al. prepared an alginate-coated chitosan nanoparticle encapsulated Fe_3_O_4_-saturated BLF (FebLf NCs) and used it in MDA-MB-231 cells and a mouse tumor model (Kanwar et al., 2016). The results showed that FebLf NCs had a spherical appearance with a particle size of only 80 ± 5 nm. Furthermore, FebLf NCs exhibited antiproliferative effects against breast cancer cells, decreased cell viability, inhibited clone formation, reduced the diameter of 3D tumor spheroids, enhanced mitochondrial depolarization, reduced tumor volume in mice, and activated the pro-apoptotic protein of caspase-3, which was associated with the activation of the P13K signaling. However, this study did not compare the anticancer effects of LF nanoparticles with different trivalent iron ion saturation. This question was addressed by Roy et al., who successfully designed two LF nanoparticles with different iron saturations, one was apo-bovine lactoferrin (Apo-bLf) (∼ 2% iron saturated)-encapsulated Eri silk nanoparticles (NPs) (Apo-bLf-loaded silk NPs) and another was Fe-bLf (100% iron saturated)-encapsulated Eri silk nanoparticles (Fe-bLf-loaded silk NPs), and used them to treat breast cancer (Roy et al., 2016). The two nanoparticles did not differ significantly in terms of particle size, microscopic image, molecular weight, and fourier transform infrared (FT-IR) spectra. However, both nanoparticles were internalized by breast cancer cells, with Apo-bLf-loaded silk NPs showing greater internalization compared to void NPs, which resulted in higher cell toxicity, greater caspase-3 release, and better efficacy in reducing the tumor sphere size of Apo-bLf-loaded silk NPs than Fe-bLf-loaded silk NPs. Nevertheless, qPCR and western blot results showed that Fe-bLf-loaded silk NPs exhibited comparatively higher pro-apoptotic effect, evidenced by a more significant upregulation of Survivin, Fas, Fas-L, lipoprotein receptor-related protein (LRP)1, LRP2, caspase-8, caspase-9, and caspase-3 and a more significant downregulation of epidermal growth factor receptor (EGFR), transferrin receptor (TfR) 1, and TfR2. Viewed together, these two nanoparticles represent promising anti-cancer materials, implying that Eri silk NPs have broader medical uses.

**TABLE 3:** Anti-breast cancer effects and mechanisms of dairy-based nanoparticles.

Nanoparticle	Characterization	Model	Anti-breast cancer effects	Pathway	References
HP, HP-C8, HP-C10 and HP-C12	Average diameter: 10–20 nm	MCF-7 cells were added with 20, 40, 60, 80 and 100 μM nanoparticle	Cell cytotoxicity, E-cadherin, Bax, caspase-8↑; G2/M phase↑; migration↓	Induced G2/M cell cycle arrest	Song et al. (2024)
LPO-loaded NPs with LF	Loading capacity: 62.3%, EE: 88.7%, desorption capacity: 96.7%, size: 477.5 nm, zeta potential: 20.6 mV	MCF-7 cells were treated with various concentrations of NPs	IC50: 150.1 ± 4.8 μg/mL, cell viability↓; apoptosis, p53↑; G0/G1 and G2/M phases arrest; NF-κB, Bcl-2↓	Induced G0/G1 and G2/M cell cycle arrest; p53 signaling↑; NF-κB signaling↓	Abu-Serie and El-Fakharany (2017)
Apo-bLf-loaded silk NPs; Fe-bLf-loaded silk NPs	Size: 200–300 nm; irregular shape	MDA-MB-231 and MCF-7 were added with NPs	MDA-MB-231: LRP1, LRP2, LFR, and TFR1↑; MCF-7: TFR and TFR2↑; cytotoxicity, apoptosis↑; Bax, caspase-9, caspase-3, caspase-8, caspase-7, TRAIL, Fas and Fas-Ligand↑; Bcl-2, survivin↓	Mitochondria mediated apoptosis pathway↑	Roy et al. (2016)
Fe-bLf NCs	Size: 80 nm ± 5 nm	MDA-MB–231 cells were treated with 2.4% w/w of FebLf NCs; mice were fed with oral administration of FebLf NCs nanoformulated diet	Cell proliferation, tumor colony, cell viability, tumor volume, tumor spheroid size, invasion↓; surviving, livin↓, cytochrome C, mitochondrial membrane depolarization ↑, LRP1, LRP 2, TFR, TFR 1, TFR 2, DMT-1, ferroportin, ferritin-heavy chain and ferritin-light chain receptor genes, caspase-3↑; tumor volume↓	PI3K signaling↑	Kanwar et al. (2016)
LF-HMP NPs	Size: 280.8–799.6 nm; SEM, globular shapes with nano-scale size	MCF-7 cells were treated with 500 μL NPs	Cell viability↓		Aval et al. (2021)
PMT/HK-loaded Lf-CMC NGs	Size: 123.0–273.1 nm; PDI: 0.230–0.389, zeta potential: −14.7 to −41.2 mV	MDA-MB-231 breast cells were added with 20, 40, 60, 80 and 100 μg/mL NPs; mice was injected with 2.5 PMT mg/kg was injected with NPs 3 times per week for 21 days	Cytotoxicity, active caspase-3, caspase-8, caspase-9, apoptosis↑, VEGF-1, tumor size, Ki67↓		Atallah et al. (2022)
Lf-MSNPs-PMT/EA	Size: 250.9–300.9 nm, zeta potential: −2.41–20.9 mV, PDI: 0.226–0.778, entrapment efficiency: 86.1%–88.5%	MCF-7 cells were added with 10, 20, 30, 40, 50, 60, 70, 80, 90, and 100 μg/mL of NPs	Cell viability↓; cellular uptake↑		Ali et al. (2020)
AuNP-BLG-CUR/GEM	Size: 29.0–41.0 nm; zeta potential: −24.5 to −7.89 mV	MCF-7 cells were added with 1, 10, 100, 1,000 and 10,000 nM of NPs	Cell viability↓		Waghmare et al. (2022)
CyWD	Size: 358.05–562.3 nm, PDI: 0.1215–0.376; zeta potential: −29.66–55.785 mV, EE: 48.24%–84.58%	BALB/c mice were injected with 4T1 cells and administered with 10 mg/kg CyWD	Tumor growth↓; Dox levels, tumor damage area↑		Singh et al. (2022)
Wh@AuNPs	Anisotropic nanoparticles with various morphology	MCF-7 cells were treated with Wh@AuNPs at different times (15, 30 and 45 min)	Cell viability↓; destroyed cells↑		Borghei and Hosseinkhani (2022)
NR-CCS NPs	Hydrodynamic diameter: 16 ± 3 nm; zeta potential: −24.08 ± 2 mV; Particles are found to be spherical with a diameter of 75–80 nm with uniform distribution	4T1 cells were added with 0.5, 1, 2, 3, 6, 9, 12, 15, and 20 μg/mL CCS NPs	Cell viability, mitochondrial membrane potential, colony growth, migration, PARP↓; cellular uptake, cell death, DNA damage, ROS, CHOP, γ-H2AX, G2/M phase↑; cell death (80% ± 4%)	Lysosome-mediated autophagic pathway; induced G2/M cell cycle arrest	Khatun et al. (2023)
Cur-CasNPs	Drug content: 8.02 μg/mg; diameter: 1–10 nm; average hydrodynamic diameter (H_d_): 225 nm; zeta potential: −20 mV	MCF-7 cells were added with 5, 10, 20, 30, 40 and 50 μM Cur-CasNPs	Cytotoxicity↑		Barick et al. (2021)
CCNG NPs	EE: 67%; hydrodynamic diameter: 160 nm; PDI: 0.130–0.177, zeta potential: −2.53 ± 3 mV; spherical particles ranged in size from 85 to 101 nm	4T1 cells were incubated with 3 μg/mL of CCNG NPs; 4T1 BALB/c mice model were injected with CCNG NPs	Cellular uptake, cytotoxicity, dead cell, ROS, ɣ H2AX, p53, DNA damage, apoptosis, cell deaths↑; mitochondrial membrane potential, invasion, migration, colony formation↓; tumor size↓; accumulation of dead cells and fluids, neutrophil infiltration, fibroblastic activity↑; spleen enlargement, mild to moderate hypertrophy of lymphatic follicles with hyperplasia of lymphocytes		Khatun et al. (2024)

HP, casein bioactive peptides nano-assemblies; HP-C8, C8 fatty acid modified-casein bioactive peptides nano-assemblies, HP-C10, C10 fatty acid modified-casein bioactive peptides nano-assemblies; HP-C12, C12 fatty acid modified-casein bioactive peptides nano-assemblies; SEM, scanning electron microscopy; LPO-loaded NPs, with LF, bovine lactoperoxidase-loaded chitosan nanoparticles with lactoferrin; Apo-bLf-loaded silk NPs: biodegradable Eri silk nanoparticles loaded with apo-bovine lactoferrin (Apo-bLf) (∼2% iron saturated); Fe-bLf-loaded silk NPs: biodegradable Eri silk nanoparticles loaded with Fe-bLf (100% iron saturated); LRP: lipoprotein receptor-related protein; LFR, lactoferrin receptor; TFR: transferrin receptor; EGFR, epidermal growth factor receptor; BAX, bcl-2-like protein 4; TRAIL, tumor necrosis factor (TNF)-related apoptosis-inducing ligand; P13K, phosphatidylinositol 3-kinase; CXCR7, chemokine receptor 7; Dox, doxorubicin; Fe-bLf NCs, Fe3O4-saturated bovine lactoferrin nanocapsules; DMT, divalent metal ion receptors; LF-HMP NPs, lactoferrin loaded-pectin nanoparticles; PMT/HK-loaded Lf-CMC NGs, antimetabolite pemetrexed (PMT)/herbal polyphenol honokiol (HK)-loaded lactoferrin-polysaccharide carboxy methyl cellulose nanogels; Lf-MSNPs-PMT/EA, lactoferrin-coupled mesoporous silica nanoparticles containing cytotoxic drug pemetrexed or the phytomedicine ellagic acid; AuNP-BLG-CUR/GEM, β-lactoglobulin conjugated gold nanoparticles containing curcumin and gemcitabine; CyWD, N-acetyl cysteine modified-doxorubicin hydrochloride-loaded genipin-crosslinked whey protein nanoparticles; Wh@AuNPs, Au and whey protein-based nanoparticles containing trypan blue; NR-CCS NPs, 10-Hydroxycamptothecin and nile red-encapsulated casein nanoparticles; ROS, reactive oxygen species; PARP, Poly (ADP, ribose) polymerase; CHOP, C/EBP, homologous protein; Cur-CasNPs, curcumin encapsulated casein NPs; CCNG NPs, glutathione-IR, 797 coupled casein nano-trojan; PDI, polydispersity index.

Considering the need to enhance therapeutic effects and decrease the toxicity of chemotherapeutic agents, several studies have integrated these chemotherapeutic agents into LF-modified nanomaterials. Current research trends favor the use of dual or triple drug combinations to enhance the anti-breast cancer efficacy of LF nanoparticles. Atallah et al. developed crosslinked LF-carboxymethyl cellulose nanogels (Lf/CMC NGs) to encapsulate PMT and the herbal polyphenol honokiol (HK), achieving dual-drug therapy for breast cancer (Atallah et al., 2022). The nanoparticles showed an HK EE of 66.67% and a PMT conjugation efficiency of 80.1%, demonstrating excellent serum stability, low hemolytic activity, and can be effectively taken by breast cancer cells. This led to anti-breast cancer effect through the inhibition of angiogenesis, promotion of apoptosis in cancer cells, and increased expression of apoptotic proteins. However, the study did not explore the mechanisms behind the anti-breast cancer effects of LF-modified NPs. The triple drug combination represents an evolution of the dual drug strategy, aimed at maximizing anti-breast cancer efficacy through synergistic interactions among three different drugs. Salah et al. created triple drug-loaded nanoparticles by combining pemetrexed (PMT), rosuvastatin (RST), and HK within sodium alginate (ALG) and LF nanohybrids, achieving loading efficiencies of 7.86% RST, 5.24% PMT, and 6.11% HK (Salah et al., 2022). These nanoparticles showed prolonged drug release, stability in blood and serum, and significant cytotoxicity against breast cancer cells, with an IC_50_ of 0.94 µM for MCF-7 cells, a 5-fold reduction in tumor size after 3 weeks of treatment, and enhanced active caspase-3 levels alongside reduced VEGF and Ki67 expression. Interestingly, certain studies have introduced magnetic fields and photothermal therapy into LF nanoparticles loaded with chemotherapeutic drugs, not only improving anti-breast cancer efficacy and reducing treatment duration but also lowering tumor recurrence risk and mitigating chemotherapy resistance. Lf-Doxo-MMNPs (Size: 130 ± 1.48 nm), a therapeutic NP integrating chemotherapy, magnetic field, and photothermal tools, demonstrated prolonged blood circulation as well as notable cytotoxicity in breast cancer cells/tissues by activating Bax and caspase-3 and modulating CXCL12 and CXCR7, thereby causing necrosis and apoptosis, blocking cell cycle at the S1 and subG1 phases, and inducing ROS accumulation (Sharifi et al., 2020). Future investigations should focus on optimizing LF-modified nanoparticle formulations or combining diverse therapeutic strategies with nanoparticles to maximize efficacy while minimizing or avoiding the side effects of chemotherapy.

β-Lactoglobulin (BLG) is another commonly used material for preparing anticancer nanoparticles. It has a β-barrel structure consisting of eight anti-parallel β-sheets with high colloidal stability and net charge distribution, which facilitates the formation of a tight structure with other materials for delivering active substances to targeted sites (Salah et al., 2020; Gao et al., 2021). In one study, AuNP-BLG-CUR and AuNP-BLG-GEM were prepared by conjugating β-lactoglobulin (BLG) with gold nanoparticles (AuNP) and embedding curcumin (CUR) or gemcitabine (GEM) into it, which effectively prevented the degradation of curcumin due to light and oxidation. The particle size of AuNP-BLG ranged from 29 to 41 nm, with a zeta potential of −24.5 to −7.89 mV. The MTT results showed that the IC_50_ values of AuNP-BLG against MCF-7 cells were 1.445 nM at 24 h and 0.863 nM at 48 h, showing a very strong anticancer effect. However, the drawback of this study is that the anticancer effects and anticancer mechanisms of AuNP-BLG-CUR and AuNP-BLG-GEM were not further revealed in an *in vivo* model.

Whey protein, a by-product of cheese processing, is not only nutritionally valuable but also exhibits good availability, high resistance to pepsin digestion, and excellent protective film-forming abilities (Khan et al., 2019; Liu et al., 2022). In addition, whey protein is also thermoprotective compared to other materials as it resists heating-induced increase in flexible conformation and exposure of hydrophobic fragments on its exterior (Liu et al., 2022). In a study employing whey protein nanoparticles for the treatment of breast cancer, N-acetyl cysteine-modified doxorubicin hydrochloride-loaded whey protein nanoparticles (CyWD) were successfully synthesized and exhibited a particle size of 358.05–562.3 nm, a PDI of 0.1215–0.376, a zeta potential of −29.66–55.785 mV and an EE of 48.24%–84.58% (Singh et al., 2022). The optimally formulated nanoparticles were used in mice and results revealed that the tumor volume, areas of tumor damage and concentration of Dox significantly (*p* < 0.05) decreased after administration of CyWD, suggesting that CyWD greatly improved drug delivery efficiency and enhanced tumor suppression. However, this study lacks mechanistic exploration reagrding tumor inhibition by CyWD. Furthermore, although the formulation of CyWD is well-designed, its efficacy *in vivo* warrants further investigation.

Casein constitutes 80% of milk protein, with approximately 95% existing in the form of colloidal particles (Zimet et al., 2011). These particles are usually spherical, with diameters ranging from 50 to 500 nm (average 150 nm) and masses ranging from 10^6^ to 3 × 10^9^ Da (average 108 Da), rendering them extremely suitable for nanoparticles preparation due to their strong tendency to associate and very high loading capacity (Zimet et al., 2011). Moreover, another advantage of casein is that it lacks disulfide bonds, contains abundant content of proline, and has poor secondary and tertiary structure, making it an intrinsically unstructured protein. This characteristic allows for easy chemical modification, further enhancing the performance of the nanoparticles (Gandhi and Roy, 2019). Recently, casein nanoparticles have been used to enhance the DNA-damaging ability of 10-hydroxy camptothecin (CPT) on cancer cells. In a study of Khatun et al., CPT and Nile red-encapsulated casein nanoparticles (NR-CCS NPs) were synthesized, achieving an EE of 77.47%–80.79%, a hydrodynamic diameter of 16 ± 3 nm, a zeta potential of 24.08 ± 2 mV, and a spherical appearance (Khatun et al., 2023). NR-CCS NPs exhibited the highest CPT release efficiency under simulated tumor microenvironment conditions (pH 5.5°C and 37°C), with a mucin binding efficiency of 80% ± 2%. These NPs demonstrated excellent biocompatibility and intracellular uptake ability, which led to decreased 4T1 cell activity, increased apoptosis, inhibited clone formation, reduced cancer cell migration ability, elevated mitochondrial membrane potential, cell cycle arrest in the G2/M phase, aggravated DNA damage, and increased ROS accumulation. Further studies showed that these changes were related to the activation of the lysosome-mediated autophagy pathway. Based on previous studies, Khatun et al. improved the targeting ability of the nanoparticles for triple-negative breast cancer by creating glutathione (GSH)-IR 797 and CPT coupled casein nano-trojan (CCNG NPs), which can naturally bind to the overexpressed gamma-glutamyl transpeptidase (GGT) in cancer cells due to the presence of GSH, thus allowing for precise targeting of cancer cells (Khatun et al., 2024). The hydrodynamic diameter, PDI and zeta potential of the CCNG NPs were 160 nm, 0.130–0.177, and −2.53 ± 3 mV, respectively. The scanning electron microscopy experiments revealed that CCNG NPs had a larger volume than NR-CCS NPs, and accordingly, some parameters also changed. The killing effect of CCNG NPs against 4T1 cells was enhanced, reaching up to 85% ± 8.5%, alongside significant increases in ROS levels and mitochondrial membrane potential. Besides, this study also found that tumor volume and mice weight decreased significantly (*p* < 0.05) after 7 days of CCNG NPs injection, and a large number of cancer cell necrosis, and neutrophil infiltration were observed. Interestingly, an inhibitory effect on spleen enlargement was also observed in the CCNG NPs group, suggesting that CCNG NPs may be involved in immunomodulation, although this hypothesis was not confirmed in this study. Both two studies are well-established, in-depth studies exploring nanoparticle characterization, *in vivo* release and cancer inhibition. In the long term, there have been some basic research explorations on dairy protein-based nanoparticles in the field of breast cancer, but clinical studies on dairy protein-based nanoparticles are still lacking. Moreover, explorations such as optimizing the formulation of dairy protein nanoparticles, enhancing the encapsulation ability of milk protein nanoparticles, and improving the therapeutic effect of nanoparticles, are worthy of further development. Finally, the exploration of the anticancer mechanism of some dairy protein-based nanoparticles, such as β-LG nanoparticles and whey protein nanoparticles, is still incomplete, and these require the joint efforts of scholars. Addressing these scientific and practical issues will advance the field of milk protein nanoscience and significantly impact the progress of precision medicine, enabling the design of safe and efficient dosages of nanoparticles for anti-breast cancer drug delivery applications.

## 5 Anti-cancer effects of dairy proteins in clinical practice

Dairy products and their derivatives supply essential micronutrients, macronutrients, and probiotics, including proteins, lipids, vitamin D, calcium, potassium, and magnesium, all of which contribute to metabolic health (Marangoni et al., 2019; Hirahatake et al., 2020). Numerous studies summarize substantial evidence indicating that long-term consumption of dairy products is safe, with most associations between dairy intake and health being beneficial. These benefits extend to individuals across all age groups, expect for specific conditions such as lactose intolerance or milk protein allergy (Mihic et al., 2016; Marangoni et al., 2019). Furthermore, a study assessing the clinical safety of dairy products found that subjects consuming ½ cup of novel milk protein peptide and ½ cup of milk daily for 6 weeks did not exhibit any abnormal clinical blood markers or adverse effects; instead, they showed improvements in insulin sensitivity and the neutrophil-to-lymphocyte ratio (Kreider et al., 2011). These studies have encouraged researchers to investigate the potential health-promoting effects of dairy products and their derivatives in clinical settings, particularly regarding their impact on cancer.

Considering that dairy products have demonstrated breast cancer inhibitory effects in both *in vitro* and *in vivo* experiments, some researchers have also explored the breast cancer inhibitory ability of dairy products in clinical settings (Table 4). For instance, Bahadoran et al. investigated 100 breast cancer patients and 175 healthy individuals and found that dairy intake, including low-fat and fermented dairy products, was positively associated with a reduced risk of breast cancer (Bahadoran et al., 2013). Similar results were reported in studies by Yu et al., which involved 1,286 breast cancer patients and 1,461 controls, and Wu et al., which included more than 1 million women followed for 8–20 years (Yu et al., 2019; Wu et al., 2021). The breast cancer preventive effect of kefir, yogurt and cheese have also been revealed in the clinic. Vantveer et al. stated that compared to 289 population controls, 133 incident breast cancer cases, regardless of younger (25–44 years) or older (55–64 years), had lower consumption levels of yogurt, buttermilk, curds, Gouda cheese, and total fermented dairy products, resulting in an increased risk of breast cancer incidence (Vantveer et al., 1989). Clinical studies providing dairy-derived active peptides to patients with breast cancer are extremely rare. The only one providing dairy-derived active peptides to 134 women with breast cancer and 267 controls without breast cancer resulted in a reduced risk of ER/PR/HER2-negative breast cancer in Iranian women (Jabbari et al., 2022). However, more research evidence is needed to clarify the relationship between dairy-derived active peptides and the risk of breast cancer.

**TABLE 4:** Anti-breast cancer effects of dairy or dairy-derived peptides in clinical trials.

Treatment	Participants	Anti-breast cancer effects	References
Bioactive peptides from dairy products	134 women with breast cancer and 267 cancer-free controls	Milk-derived bioactive peptides negatively associate with the risk of ER/PR/HER2 negative breast cancer among Iranian women	Jabbari et al. (2022)
Kefir	Participants were 40 years of age and had undergone chemotherapy and/or radiation therapy within the last 2 years; The kefir group was asked to ingest 8 ounces of low-fat kefir after each exercise session during the 12 weeks intervention	Beck depression Inventory, total piper fatigue score, gastric distress score, intermediate monocytes, IL-6↓	Smoak et al. (2021)
Milk	97 patients presenting with a histopathologic diagnosis of breast cancer and 104 control individuals	High milk consumption increased the breast cancer risk by 7.2 times	Galván-Salazar et al. (2015)
Milk	93,306 participants, aged 40–69 years were included, with 359 breast cancer cases being diagnosed after 6.3 years follow-up	milk consumption in Korean women aged 50 or younger is associated with a decreased risk for breast cancer, when compared to those who never or rarely consumed milk	Shin et al. (2020)
Yogurt and buttermilk	133 incident breast cancer cases and 289 population controls	Breast cancer risk decreased	Vantveer et al. (1989)
Dairy products	438 recruited cases with primary breast cancer and 438 controls	No significant association was found between dairy products intake and breast cancer risk	Zhang et al. (2011)
Dairy products	4,697 initially cancer-free women (age ≥15); 88 breast cancers were diagnosed after 25 years	No significant interactions between milk intake and time of cancer diagnosis were observed	Knekt et al. (1996)
Dairy products	52,795 North American women, 1,057 new breast cancer cases were diagnosed after 7.9 years	Higher intakes of dairy full-fat and reduced fat milk were associated with greater risk of breast cancer	Fraser et al. (2020)
Dairy products	275 women (100 cases and 175 controls)	Higher consumption of total dairy intake was accompanied with reduced breast cancer risk [odds ratio (OR) = 0.14, 95% CI = 0.04–0.38]. Similar observations were obtained for LFD, HFD, and fermented dairy products	Bahadoran et al. (2013)
Dairy products	351,041 women, 7,379 of whom were diagnosed with invasive breast cancer during up to 15 years of follow-up	No significant associations between intake of dairy products and risk of breast cancer	Missmer et al. (2002)
Dairy products	350 patients with pathologically confirmed cases of breast cancer and 700 age-matched controls	LFD intake was inversely and HFD consumption was positively associated with breast cancer. No significant association was found between yogurt and cheese consumption and breast cancer, while total milk intake was associated with a greater odds of breast cancer	Dashti et al. (2022)
Dairy products	A total of 1,286 cases of breast cancer and 1,461 controls were enrolled	There was an inverse association between the weekly frequency of dairy intake and the risk of ER+, PR+, and ER + PR + breast cancer	Yu et al. (2019)
Dairy products	88,691 women, 3,482 women with incident invasive breast cancer	No association was found between intake of dairy products and breast cancer in postmenopausal women. Among premenopausal women, high intake of LFD, especially skim/low-fat milk, was associated with reduced risk of breast cancer	Shin et al. (2002)
Dairy products	1,699 women 26–79 years of age, including 823 breast cancer cases and 876 randomly selected controls	A significant decrease in breast cancer risk was observed with increased consumption of dairy products, for premenopausal women only	Wajszczyk et al. (2021)
Dairy products	68,567 postmenopausal women, 2,855 incident cases of breast cancer were identified after 8 years	Dairy products may modestly reduce risk of postmenopausal breast cancer	McCullough et al. (2005)
Dairy products	Over 1 million women were followed for a maximum of 8–20 years across studies and 37,861 cases were diagnosed with breast cancer	Adult dairy consumption is unlikely to associate with a higher risk of breast cancer and that higher yogurt and cottage/ricotta cheese intakes were inversely associated with the risk of ER-negative breast cancer	Wu et al. (2021)
Dairy products	1941 women diagnosed with breast cancer and 1,237 control participants	Total dairy intakes were associated with a non-significant 15% reduction in breast cancer risk; higher intakes of yogurt were associated with reduced risk of breast cancer and higher intakes of American, cheddar, and cream cheeses were associated with a marginally significant increased risk	McCann et al. (2017)
Dairy products	64,904 Norwegian women, 218 premenopausal and 1,189 postmenopausal incident breast cancer cases were diagnosed during follow-up	Total dairy, adult, and childhood milk consumption was not associated with either pre- or postmenopausal breast cancer risk. Premenopausal women with the highest consumption of white cheese had half the risk of breast cancer compared to those with the lowest consumption	Hjårtaker et al. (2010)
Dairy products	184 breast cancer patients and equal number of age-matched controls	An increased breast cancer risk in women were observed with higher consumption of milk; an inverse association with breast cancer risk was observed for total cheese intake when comparing highest with lowest quartiles. The protective effect of cheese was confirmed only for fresh cheese. No significant association was found for other types of dairy foods	Maliou et al. (2018)
Dairy products	319,826 women, with 7,119 women being diagnosed with breast cancer after 8.8 years follow-up	No clear associations of breast cancer with milk emerged	Pala et al. (2009)

LFD, low-fat dairy; HFD, high-fat dairy.

Recognizing the health benefits of dairy products, researchers have suggested providing dairy to breast cancer patients undergoing chemotherapy to promote their recovery. One clinical study offered kefir to breast cancer patients who had received chemotherapy and/or radiation within the past 2 years and found that the patients’ Beck depression inventory, total piper fatigue score, gastric distress score, intermediate monocytes, and IL-6 levels decreased significantly (*p* < 0.05), suggesting an improvement in the health status of patients (Smoak et al., 2021). Another study also supports the advantages of dairy consumption for breast cancer survivors. Research indicated that the intake of fermented dairy products reduced mortality and cancer recurrence risk among breast cancer survivors, and significantly improved disease-free survival (*p* = 0.001) and overall survival (*p* = 0.004). These studies collectively suggest that breast cancer patients receiving chemotherapy can greatly benefit from increased consumption of dairy products, particularly fermented dairy products.

Some scholars stated that the consumption of dairy products with differing fat contents may have varying effects on breast cancer. Low-fat dairy products are often associated with a reduced risk of breast cancer, while high-fat dairy products may negatively impact breast cancer development. Dashti et al., in a study involving 350 breast cancer patients and 700 healthy individuals, found that low-fat milk intake decreased breast cancer risk, whereas high-fat milk intake increased it (Dashti et al., 2022). Similarly, Shin et al. reported that high intake of low-fat dairy products, especially skim/low-fat milk, was associated with a reduced risk of breast cancer, while the consumption of high-fat dairy food and whole milk exhibited statistically nonsignificant inverse associations with premenopausal breast cancer risk (Shin et al., 2002). Further, the type of dairy product also affects the risk of breast cancer. Yogurt, fermented milk, and certain types of cheeses may be more effective than milk in reducing the risk of breast cancer. A study involving 64,904 Norwegian women, and 218 premenopausal and 1,189 postmenopausal breast cancer patients, showed that premenopausal women with the highest consumption of white cheese had a 50% lower risk of breast cancer compared to those with the lowest dairy consumption. However, other dairy intake did not show a positive or negative association with breast cancer risk in both premenopausal or postmenopausal women (Hjårtaker et al., 2010). It has also been suggested that increased yogurt intake reduces breast cancer risk, while higher consumption of American cheese, cheddar cheese, and cream cheese may increase it (McCann et al., 2017). Similarly, Maliou et al. also revealed that only the consumption of fresh cheese was associated with a reduced breast cancer risk in a study involving 184 breast cancer patients and an equal number of age-matched controls (Maliou et al., 2018). Generally, premenopausal intake of dairy products is more effective in intervening in breast cancer progression than postmenopausal intake of dairy products. For example, a case study involving 1,699 women (26–79 years of age, including 823 cancer cases and 876 randomly selected controls) indicated that higher consumption of dairy products significantly decreased breast cancer risk, but only among premenopausal women (Wajszczyk et al., 2021). This is further supported by Shin et al., who found that high low-fat dairy product intake in premenopausal women, especially skim/low-fat milk, was associated with a reduced risk of breast cancer (Shin et al., 2002).

However, several studies also claim that breast cancer risk is not associated with dairy product intake. Two of these studies involving large samples (a 15-year follow-up study of 351,041 women with 7,379 breast cancer patients, and an 8.8-year follow-up study of 319,826 women with 7,119 breast cancer patients) concluded that there was no significant association between dairy product intake and increased breast cancer risk (Missmer et al., 2002; Pala et al., 2009). Furthermore, other two studies, one involving 4,697 initially cancer-free women (88 women were eventually diagnosed with breast cancer), and another including 438 breast cancer cases and 438 controls, yielded similar results, both indicating no significant association between dairy intake and breast cancer risk. Interestingly, contrary results were reported in a study, which reported that high consumption of milk increased the risk of breast cancer by 7.2 times, but this study had a relatively small sample size, involving only 97 patients diagnosed with breast cancer and 104 controls (Galván-Salazar et al., 2015). Collectively, studies supporting the view that dairy intake has no negative effect on breast cancer are in the majority.

## 6 Conclusion, limitations, and future perspectives

Dairy products and dairy-derived peptides have exhibited notable advantages in the administration of breast cancer, such as high nutritional value, desirable efficacy, low side effects, and high compliance, which have garnered increasing scholarly attention. Different dairy product forms, containing dairy protein-derived peptides, dairy products, and dairy-protein based nanoparticles, have demonstrated anti-breast cancer proliferation, migration and invasion, pro-apoptotic and cell cycle blocking effects in both *in vivo* and *in vitro* models. Clinical trials further suggest that dairy intake is probably associated with reduced risk of breast cancer. This review provides ideas for future research and industrial application of dairy-derived peptides, dairy products and dairy-protein based nanoparticles in the fight against breast cancer. Specifically, from the perspective of the food industry, this study promotes the development of anticancer functional foods; from the perspective of nanomaterials field, this review points out problems that exist in the dairy protein-based nanomaterials field and guides new research directions for synthesizing high-performance materials; from the perspective of medical field, milk protein-derived peptides, fermented dairy products, and milk protein nanoparticles meet the patient’s needs for natural medicines that are highly efficacious, have fewer side effects, and are less expensive, presenting a promising avenue for cancer adjuvant therapy. However, due to a lack of relevant research, this review neglected to summarize the anti-breast cancer effects of less common dairy protein-derived peptides and dairy products (e.g., horse milk-derived peptides, buffalo milk yogurt, mozzarella cheese, and cheddar cheese), nor did it provide a deep discussion of the anticancer mechanisms of certain dairy products.

To address these gaps, we propose focusing on the following issues:

1. Exploration of alternative dairy sources: Deer milk, horse milk, and donkey milk may show greater anti-breast cancer effects and nutritional value. Isolating anticancer active peptides from these dairy products by enzymatic hydrolysis or fermentation is a promising research direction.
2. Active peptide identification: Fermented milk, yogurt and different types of cheese contain a large number of active peptides. Identifying highly anticancer active peptides and examining the relationship between their primary and secondary structures and anti-breast cancer activity will promote the development of small molecule peptide functional foods.
3. Mechanistic studies: The underlying mechanisms of certain dairy-derived peptides and dairy protein-based nanoparticles in combating breast cancer are not yet well understood and need further investigation. Addressing these issues will facilitate the production of functional dairy products with anticancer properties and promote their application in clinical settings.
